# Rehabilitation Nutrition 3.0—the Dissemination and Evolution of Comprehensive and Individualized Rehabilitation Nutrition: A Position Paper by the Japanese Association of Rehabilitation Nutrition (Secondary Publication)

**DOI:** 10.31662/jmaj.2026-0180

**Published:** 2026-07-24

**Authors:** Hidetaka Wakabayashi, Keisuke Maeda, Shinjiro Miyazaki, Mariko Nakamichi, Dai Fujiwara

**Affiliations:** 1 Department of Rehabilitation Medicine, Tokyo Women’s Medical University Hospital, Tokyo, Japan; 2 Nutrition Therapy Support Center, Aichi Medical University Hospital, Nagoya, Japan; 3 Rehabilitation Center, KKR Takamatsu Hospital, Takamatsu, Japan; 4 Department of Pharmacy, Haradoi Hospital, Fukuoka, Japan; 5 Department of Rehabilitation Medicine, Saka General Hospital, Shiogama, Japan

**Keywords:** physical, psychological, rehabilitation nutrition, social, spiritual, technological

## Abstract

Rehabilitation nutrition 3.0 is a comprehensive and individualized approach that maximizes the functioning and well-being of both individuals and healthcare providers by incorporating physical, psychological, social, spiritual, and technological aspects. Rehabilitation nutrition 1.0 refers to the period from 2010 to 2017, when the concept of rehabilitation nutrition was proposed in Japanese books and literature. Rehabilitation nutrition 2.0, spanning 2017 to 2025, enabled the practice of higher-quality rehabilitation nutrition through the rehabilitation nutrition care process. However, earlier approaches primarily focused on improving physical function and activity or preventing deterioration. Recently, the importance of rehabilitation nutrition has expanded beyond physical considerations to encompass psychological, social, and spiritual aspects. In addition, the rapid advancement of cutting-edge technologies, such as artificial intelligence, holds the potential to dramatically enhance both the quality and quantity of rehabilitation nutrition. Based on the principles of rehabilitation nutrition 2.0, rehabilitation nutrition 3.0 is expected to evolve into a more comprehensive, individualized approach that extends beyond physical aspects by integrating insights from not only medicine and nutritional science but also the humanities, social sciences, and information science.

This position paper was authored and approved by the Japanese Association of Rehabilitation Nutrition.

## Introduction

Rehabilitation nutrition is applicable to people of all ages living with illness, frailty, or disability. In Japan’s rapidly aging society, achieving greater well-being while living with illness or disability is a critical challenge. Rehabilitation nutrition has developed primarily in Japan as an academic and clinical field integrating rehabilitation and nutritional improvement to address this challenge. Rehabilitation nutrition 3.0 proposed in this paper is a comprehensive, individualized approach that integrates physical, psychological, social, and spiritual dimensions. Specifically, it leverages technologies such as digital health to optimize individual functioning and enhance the well-being of both individuals and healthcare providers. Artificial intelligence (AI) is one component of this field.

Rehabilitation nutrition, first proposed as rehabilitation nutrition 1.0 with the publication of Japanese books ^(1), (2)^ in 2010, has rapidly spread as a concept originating in Japan. Rehabilitation nutrition 2.0, established in 2017, introduced a unique framework called the rehabilitation nutrition care process ^(3), (4)^ to enable the practice of higher-quality rehabilitation nutrition. In 2017, rehabilitation nutrition was further defined as a process that (1) holistically evaluates functioning based on the International Classification of Functioning, Disability and Health, as well as the presence and causes of nutritional disorders, sarcopenia, and nutrient excess or deficiency (2) conducts rehabilitation nutrition diagnosis and rehabilitation nutrition goal setting; and (3) elicits the highest body functions, activities, participation, and quality of life by improving nutritional status, sarcopenia, and frailty through “nutrition care management in consideration of rehabilitation” and “rehabilitation in consideration of nutrition” in individuals with disabilities and frail older adults ^(3), (4)^. This definition explicitly states that rehabilitation nutrition is not solely about the use of oral nutritional supplements for individuals undergoing exercise or functional training. However, a framework that systematically integrates nonphysical factors, such as personal psychological conflict, social isolation, and spiritual well-being—all of which influence rehabilitation outcomes and nutritional intake—has been lacking.

Rehabilitation nutrition 2.0 is rapidly developing as an academic and clinical field originating in Japan, and its social significance continues to increase. Between 2017 and 2025, reimbursement items related to rehabilitation, nutrition, and oral management were established in both medical and long-term care reimbursement systems, reflecting the robust clinical and policy implementation of this concept (Table 1 ^(5), (6), (7)^). Although policy recognition has promoted the dissemination of rehabilitation nutrition, more rigorous academic research is needed to validate the outcomes of the rehabilitation nutrition 3.0 framework. To date, however, rehabilitation nutrition has primarily aimed at optimizing physical function and activity or preventing functional decline.

**Table 1.** Advances in Rehabilitation Nutrition 2.0 (2017–2025).

Medical fees	Additional fees for rehabilitation-nutrition–oral management coordination systems in acute care wards, placement of registered dietitians in convalescent rehabilitation wards, establishment of community-based integrated care wards, and rehabilitation-nutrition–oral management coordination fees in community-based integrated care wards.
Long-term care fees	Strengthened requirements for nutrition and oral management under the rehabilitation management fee.
Academic foundation	Hosting academic conferences by the Japanese Association of Rehabilitation Nutrition, publishing academic journals, establishing the Rehabilitation Nutrition Specialist certification system, developing Rehabilitation Nutrition Clinical Practice Guidelines ^(5)^, and creating position papers, etc.
Interdisciplinary collaboration	Joint position papers on sarcopenia and dysphagia ^(6)^ and respiratory sarcopenia ^(7)^ by four societies and joint sessions with other societies at academic conferences

Recently, the importance of rehabilitation nutrition has expanded beyond physical considerations to encompass psychological, social, and spiritual aspects. At the core of rehabilitation nutrition 3.0 is the holistic perspective that nutrition and physical function are inseparable from the psychological, social, and spiritual aspects of human beings. A comprehensive approach to rehabilitation nutrition is therefore necessary to overcome complex clinical challenges because the traditional, compartmentalized model addresses these aspects as isolated issues. For example, psychological well-being and social connections, such as dining together, can increase appetite and oral intake, thereby improving nutritional status. Additionally, proper nutritional management and rehabilitation can restore physical function, increase self-efficacy, promote social participation, and, ultimately, lead to a rediscovery of meaning in life. Furthermore, the rapid advancement of cutting-edge technologies holds the potential to enhance both the quality and quantity of rehabilitation nutrition. In our vision for the future of the field, we anticipate that rehabilitation nutrition will evolve beyond its traditional physical focus to embrace the transformative potential of technology. Rehabilitation nutrition 3.0 is similar to palliative care in that both provide comprehensive care. However, it differs from existing care models in that it incorporates these elements into the clinical reasoning cycle of the rehabilitation nutrition care process. This paper clarifies the position of the Japanese Association of Rehabilitation Nutrition in pursuing a more prosperous and equitable society for both individuals and healthcare providers through rehabilitation nutrition 3.0—a comprehensive, individualized approach that integrates these multifaceted elements.

## Rehabilitation Nutrition 3.0 Integrated Framework

The rehabilitation nutrition 3.0 integrated framework encompasses six dimensions: physical, psychological, social, spiritual, technological, and healthcare provider well-being (Table 2 ^(8), (9), (10), (11), (12), (13), (14), (15), (16), (17), (18)^; Figure 1). Through this six-dimensional approach, rehabilitation nutrition 3.0 aims to maximize individuals’ functioning and well-being while positing that enhancing the well-being of healthcare providers directly contributes to improving the well-being of the individuals they serve. The following subsections provide an overview of each aspect.

**Table 2.** The Integrated Framework of Rehabilitation Nutrition 3.0.

Physical aspects	Improving functioning and quality of life through the rehabilitation nutrition care process; nutritional physical therapy ^(8), (9)^; rehabilitation pharmacotherapy ^(10), (11)^; rehabilitation-nutrition–oral management collaboration ^(12), (13), (14)^; and the prevention and treatment of malnutrition, sarcopenia, and cachexia.
Psychological aspects	Supporting mental health and behavioral change through, exercise therapy, nutritional therapy, psychotherapy, social support, and other nonpharmacological approaches for depression prevention and mental health improvement ^(15)^; positive psychology and rehabilitation nutrition ^(16)^.
Social aspects	Health disparities and promoting social inclusion ^(17)^; social isolation; loneliness; economic hardship; living environment; social determinants of health; social activities at micro- (individual), meso- (community), and macrolevels (policy/international); ultraprocessed foods.
Spiritual aspects	Supporting life’s meaning and dignity; spiritual pain; approaches to temporality, relationality, autonomy, and independence; active listening and empathy; presence; music therapy, art therapy, aromatherapy, bibliotherapy, journaling ^(18)^.
Technological aspects	Artificial intelligence, virtual reality and augmented reality, telemedicine, wearable devices, digital health, data science (including omics information such as genetic data, proteomics, metabolomics, and microbiome data).
Healthcare provider well-being	Consideration for healthcare providers’ physical, psychological, social, spiritual, and technological aspects; improving work efficiency; preventing burnout; learning and enhancing expertise; facilitating smooth interdisciplinary collaboration.

**Figure 1.**
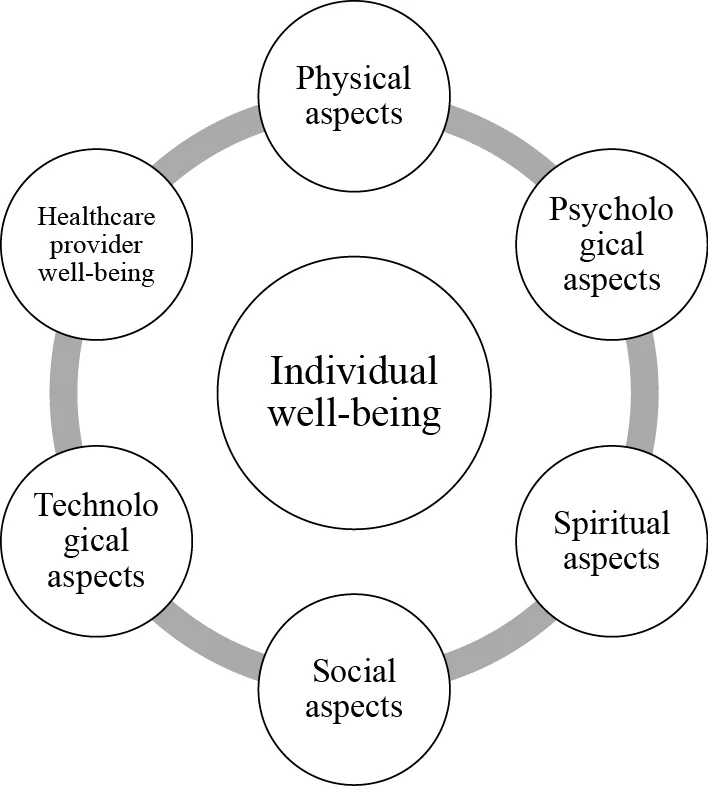
The integrated framework of rehabilitation nutrition 3.0. Rehabilitation nutrition 3.0 maximizes the functioning and well-being of individuals by addressing six dimensions: physical, psychological, social, spiritual, technological, and healthcare provider well-being.

### Physical aspects

The rehabilitation nutrition care process is also applied in rehabilitation nutrition 3.0. However, we should move beyond the traditional medical model focused on identifying and addressing weaknesses and instead incorporate the perspective of positive psychology that identifies and leverages an individual’s strengths ^(16)^. For example, among patients diagnosed with malnutrition according to the Global Leadership Initiative on Malnutrition criteria—such as those experiencing unintended weight loss without low body mass index or muscle mass loss—simply stabilizing body weight can often lead to enhanced functioning and well-being.

When setting nutritional goals, establishing specific targets for weight gain or loss within a defined timeframe is a practical strategy ^(19)^. Shared decision-making should guide the nutritional goal-setting process, integrating the individual’s perceived ideal weight and personal preferences rather than relying solely on the healthcare provider’s assessment of healthy and current weight. Collaborative goal-setting enhances self-efficacy, thereby improving adherence to the exercise and nutritional regimens necessary to achieve the goal. Once a weight goal is set, an aggressive nutritional therapy approach ^(20), (21)^ is implemented to achieve this goal.

Rehabilitation nutrition assessment and diagnostic reasoning involve diagnosing the causes of appetite loss, weight loss, and sarcopenia ^(22)^. While numerous causes exist, a comprehensive evaluation must prioritize not only physical diagnoses but also nonphysical factors such as cachexia, adverse drug effects, depression, spiritual pain, and social determinants, which are often neglected in traditional assessments.

The latest version of the rehabilitation nutrition clinical practice guidelines is release in 2026. These guidelines will provide interdisciplinary teams with an evidence-based rationale for implementing and promoting rehabilitation nutrition, serving as essential tools to optimize clinical practice.

### Psychological aspects

Extrapolating from findings in psychiatry, a network meta-analysis of randomized controlled trials examining the effects of exercise on depression found walking, jogging, yoga, and strength training to be more effective than other forms of exercise for depression, suggesting that exercise should be considered alongside psychotherapy and antidepressants as a core treatment for depression ^(23)^. Furthermore, moderate-to-vigorous aerobic exercise has demonstrated therapeutic efficacy not only for depression but also for social phobia, panic disorder, generalized anxiety disorder, posttraumatic stress disorder, brief psychotic disorder, schizophrenia, schizoaffective disorder, delusional disorder, schizophreniform disorder, attention-deficit/hyperactivity disorder, and neurodevelopmental disorders ^(24)^. Therefore, exercise is indispensable for addressing the psychological aspects of rehabilitation nutrition.

As outlined herein, there is evidence in the field of psychiatry linking depression and nutrition ^(25), (26), (27), (28), (29), (30), (31), (32), (33)^. A meta-analysis exploring the association between malnutrition and depression in older adults reported that malnourished individuals had a significantly higher risk of depression, with an odds ratio of 2.03 ^(25)^. Conversely, a meta-analysis exploring the link between obesity and depression reported an odds ratio of 1.33, with increasing obesity prevalence associated with a higher prevalence of depression ^(26)^. Intentional weight loss among individuals with obesity is often associated with improvements in depressive symptoms ^(27)^. Regarding micronutrients, meta-analyses have shown that intake of vitamin D ^(28)^ and zinc ^(29)^ improves depressive symptoms. These nutrients act on neurotransmitter synthesis and suppress brain inflammation. Higher dietary fiber intake is associated with lower depression, with each 5 g increase in fiber intake reducing depression risk by 5% ^(30)^. In addition, probiotic interventions have been shown to reduce symptoms of depression and anxiety ^(31)^. Probiotics are thought to be useful for alleviating depressive symptoms through anti-inflammatory effects, gut microbiota modulation, cognitive enhancement, and effects on brain structure and neurotransmitter systems ^(32)^. A systematic review examining the association between alcohol consumption and depression found a lower risk of depression when pure alcohol intake was moderate or lower (average 0-24 g/day, with consumption exceeding 48 g/day less than once per week) ^(33)^. Collectively, these findings suggest that nutrition is important for the prevention and treatment of depression.

### Social aspects

Rehabilitation nutrition 3.0 incorporates social and environmental factors. Health, nutritional status, functioning, and well-being are not determined solely by individual effort but are also strongly influenced by social determinants of health, including social isolation, economic hardship, living conditions, and community environment. For example, economic hardship leads to increased consumption of inexpensive ultraprocessed foods. These foods typically contain excessive amounts of sugar, oil, and salt while remaining poor sources of essential nutrients such as protein, vitamins, and minerals. Extrapolating from the field of public health, an umbrella review examining the link between ultraprocessed foods and health found that their consumption was associated with increased risks of reduced kidney function, wheezing in children and adolescents, diabetes, overweight, obesity, depression, general mental disorders, mortality, and sleep-related disorders ^(34), (35)^. Therefore, excessive consumption of ultraprocessed foods should be avoided. However, such foods may still play a role in texture-modified diets for individuals with swallowing disorders and in oral nutritional supplements used to improve nutritional status.

Socially isolated older adults are prone to losing motivation to prepare meals, leading to appetite loss and malnutrition. The social aspects of rehabilitation nutrition include not only microlevel approaches aimed at improving individuals’ functioning through social engagement but also mesolevel approaches involving community networks to prevent solitary eating and research groups such as rehabilitation nutrition networks, as well as macrolevel approaches advocating for healthcare policy and institutional reform. The strong emphasis on individual responsibility associated with extreme neoliberalism in contemporary Japanese society, socially isolates individuals facing hardship and intensifies feelings of loneliness. Indeed, extrapolating from social psychology, exposure to neoliberalism increases loneliness, diminishing happiness and the sense of connection with others ^(36)^. Furthermore, heightened competitive awareness toward others amplifies this loneliness and further erodes individual well-being ^(36)^. Therefore, in rehabilitation nutrition 3.0, while respecting individual effort, it is crucial to focus on these broader social contexts and adopt approaches that strengthen connections with others.

### Spiritual aspects

Rehabilitation nutrition 3.0 includes support for spiritual aspects such as the meaning and dignity of life. Spirituality does not refer solely to religious aspects but also encompasses fundamental questions and desires related to human existence, including the meaning and purpose of life, hope, dignity, values, suffering, and connection with others and the universe. Spiritual pain occurs when individuals lose sight of their sense of purpose or meaning in life because of temporality (limited future time), relationality (loss of connection with others), autonomy (loss of self-determination, freedom of choice, or sense of control), or independence (loss of the ability to perform activities of daily living [ADLs] independently). This leads to an unbearable sense of isolation, despair, worthlessness, or unjust suffering. Many individuals receiving rehabilitation nutrition experience invisible spiritual pain associated with end-of-life conditions, permanent disabilities, social environmental factors, or other forms of loss. Furthermore, in Japan’s aging society, where mortality is increasing, the number of individuals receiving rehabilitation nutrition near the end of life is rising. Rehabilitation nutrition thus represents a vital framework for supporting life with dignity and enhancing well-being throughout an individual’s remaining lifespan.

Rehabilitation nutrition 3.0 addresses spiritual pain related to temporality, relationality, autonomy, and independence, grounded in healthcare providers’ kindness, compassion, and supportive attitude toward patients ^(18)^. Specifically, spiritual care emphasizes active listening and empathy, providing reassurance that the patient is not alone through shared presence. This approach facilitates the process of rediscovering one’s own value and purpose in life, maintaining or rebuilding hope, cultivating gratitude toward others, and forgiving oneself.

Nonpharmacological therapies such as music therapy, art therapy, aromatherapy, bibliotherapy (reading therapy), and journaling (mindful writing) also serve as approaches to addressing spiritual aspects. For individuals requiring specialized knowledge or skills, collaboration with relevant professionals, including chaplains and spiritual care specialists, should be considered as needed.

### Technology aspects

Rehabilitation nutrition 3.0 requires actively leveraging rapidly evolving innovative technologies such as AI. AI, wearable devices, telemedicine, digital health, and genetic/omics information enhance the individualization and efficiency of rehabilitation nutrition.

AI is expected to be used at each stage of the rehabilitation nutrition care process, enhancing its individualization and efficiency (Table 3). During the assessment and diagnosis phase, deep learning algorithms can automate the evaluation of muscle mass using medical imaging and assist with diagnostic reasoning for appetite loss. During the goal-setting phase, machine learning models can predict personalized weight targets by analyzing integrated body composition and ADL datasets. During the intervention and monitoring phase, generative AI can propose personalized nutritional and exercise programs. Computer vision technologies can automatically estimate dietary intake from meal images, ensuring real-time adjustments. During outcome evaluation, AI can objectively evaluate the degree to which set goals have been achieved by performing a thorough analysis of accumulated data. This improves the quality of feedback for the next care cycle. Using AI is beneficial for high-quality clinical practice, research, and education in rehabilitation nutrition. The ability to use AI is an important attribute of knowledge workers. Healthcare providers, as knowledge workers, should leverage AI.

**Table 3.** Examples of AI Support for Rehabilitation Nutrition Care Processes.

Rehabilitation nutrition assessment and diagnostic reasoning, rehabilitation nutrition diagnosis	Diagnosis of malnutrition, sarcopenia, and cachexia presence; diagnostic reasoning for causes of appetite loss, weight loss, and sarcopenia - Machine learning/deep learning
Rehabilitation nutrition goal setting	Weight goal setting based on integrated analysis of body composition (muscle mass, fat mass), activities of daily living, swallowing function, and other data - Machine learning, generative AI
Rehabilitation nutrition intervention	In addition to treatment recommended by clinical practice guidelines, proposes prioritized interventions such as optimal nutrition interventions and exercise programs based on causal reasoning - Reasoning AI, generative AI Support for individualized advice and motivation maintenance through AI chatbots to encourage behavioral change - Generative AI
Rehabilitation nutrition monitoring	Automatically estimates intake volume and nutrients from meal image analysis (pre- and postmeal) to adjust dietary plans - Deep learning

AI: artificial intelligence.

Digital health is a concept encompassing mobile health, health information technology, wearable devices, telemedicine, and more, aiming to improve health and well-being through technology. Telemedicine and digital health eliminate geographic constraints, thus contributing to continuity of care and expanded access when used alongside AI, making home-based and community-based rehabilitation nutrition more feasible. Specifically, the use of digital health as a communication tool among home healthcare staff is expected to enable smooth information sharing and collaboration among interdisciplinary rehabilitation nutrition teams. Furthermore, wearable devices and smartphone apps enable real-time collection and analysis of data such as activity levels, dietary intake, and sleep patterns, facilitating personalized, data-driven approaches. Extrapolating from rehabilitation medicine, virtual rehabilitation in virtual spaces using virtual reality and augmented reality can enhance motivation for exercise and make functional recovery more enjoyable ^(37)^.

The application of data science to genetic information, proteomes, metabolomes, and microbiomes is also included in rehabilitation nutrition 3.0. This information clarifies individual characteristics related to nutrient metabolism and muscle synthesis, leading to personalized rehabilitation nutrition.

Extrapolating from emerging interdisciplinary research, a review of interdisciplinary approaches using technology for sarcopenic dysphagia highlights robotic swallowing technology, stem cell therapy, and precision medicine considering skeletal muscle genotypes ^(38)^. In the domain of digital health, a meta-analysis examining the effects of digital lifestyle interventions on depression, anxiety, and stress found moderate effects for depression and mild effects for anxiety and stress ^(39)^. Therefore, healthcare providers are required to leverage technological advancements in the practice of rehabilitation nutrition.

Seamless sharing of rehabilitation nutrition information across multiple facilities enables lifelong, continuous provision of rehabilitation nutrition. Currently, information on rehabilitation, nutrition, and psychology is often excluded from patient information sharing systems. Establishing mechanisms for information sharing, such as through the use of individual number cards, is crucial for enhancing the quality and continuity of rehabilitation nutrition.

### Healthcare provider well-being

Rehabilitation nutrition 3.0 incorporates the improvement of healthcare provider well-being. Increasing medical complexity and workloads heighten healthcare providers’ risk of burnout and depression. Burnout among nurses is globally prevalent, with high workloads and stress identified as contributing factors ^(40)^. If healthcare providers delivering rehabilitation nutrition experience physical or mental distress, delivering high-quality rehabilitation nutrition to patients becomes difficult. Low healthcare provider well-being tends to correlate with lower patient well-being. Extrapolating from the field of healthcare quality and safety, meta-analyses show that higher levels of burnout among physicians and nurses correlate with poorer healthcare and patient care quality ^(41), (42)^. Therefore, rehabilitation nutrition 3.0 positions the improvement of healthcare provider well-being as a prerequisite for enhancing the quality of patient care.

Integrating evidence from adjacent health sciences, our framework proposes that healthcare providers are encouraged to practice self-care in physical aspects such as exercise, nutrition, and sleep; psychological aspects including cognitive behavioral therapy ^(43)^ and mindfulness ^(44)^; social and spiritual aspects ^(45)^; and technological aspects such as AI use ^(46)^. Achieving a balance between professional responsibilities and personal life is fundamental to maintaining physical and mental health, ensuring adequate time for rest and recovery. While leveraging AI to enhance work productivity, efforts should focus not only on increasing output but also on expanding personal time outside of work. In addition to self-care, well-being care should also be implemented within the rehabilitation nutrition team. It is vital for team members to be mindful of each other’s physical and mental condition, detect signs of distress early, and reach out to one another.

The forms of capital supporting well-being, including but not limited to those relevant to healthcare professionals, include human capital, social capital, and financial capital, all of which mutually influence each other. Therefore, increasing these forms of capital is also crucial for healthcare providers to enhance their well-being. In rehabilitation nutrition, healthcare providers can accumulate human capital—such as knowledge, skills, experience, and qualifications related to rehabilitation nutrition—as well as social capital through participation in the rehabilitation nutrition team, the rehabilitation nutrition network study group, and the Japanese Association of Rehabilitation Nutrition.

## Challenges and Prospects for Rehabilitation Nutrition 3.0

From our perspective, the ongoing effects of excessive neoliberalism and globalization are expected to widen disparities in overall well-being across physical, psychological, social, spiritual, and technological dimensions. We posit that the Matthew effect, whereby the wealthy become wealthier while the poor become poorer, could exacerbate inequality in well-being. Addressing this issue will require a more holistic approach, such as rehabilitation nutrition 3.0. The following subsections outline the challenges and prospects of rehabilitation nutrition 3.0 in addressing these widening disparities in health and well-being.

### Building evidence in physical, psychological, social, spiritual, and technological domains

Traditional rehabilitation nutrition has primarily focused on building evidence in physical aspects. While continuing to strengthen the evidence base in physical aspects, it is crucial to simultaneously develop evidence in psychological, social, spiritual, and technological dimensions and publish these findings in peer-reviewed literature. Clinical trials should be conducted comparing groups using the rehabilitation nutrition 3.0 framework with groups using the conventional rehabilitation nutrition 2.0 model. Outcome measures should include well-being, meaning in life, and spiritual pain.

### Promoting social implementation

Leveraging the foundation of medical and long-term care reimbursement systems established by 2025, further social implementation of rehabilitation nutrition will be pursued. As proponents of this framework, we envision that, by the 2030s, basic hospitalization fees in acute-care hospitals could incorporate a collaborative system for rehabilitation, nutrition, and oral management. In this scenario, rehabilitation nutrition would transition from a specialized add-on service to a standard component of care. The goal is also to promote the spread of rehabilitation-nutrition–oral management collaboration in community and home care settings.

### Full-scale AI implementation and ethical challenges

Among the physical, psychological, social, spiritual, and technological dimensions, the technological aspects are changing most rapidly. As a future-oriented initiative within this framework, to prevent widening well-being disparities stemming differences in AI access and use, we will address ethical challenges ^(47)^ and develop/provide educational programs that enable healthcare providers and recipients to use AI appropriately. For example, online training sessions provide vital opportunities to acquire AI proficiency and integrate its application directly into the rehabilitation nutrition care process. Although economic disparities may lead to differences in AI access and use, free AI tools (such as Gemini or DeepL) should be leveraged. Healthcare providers and individuals must maintain agency over AI, ensuring that they use these tools as extensions of their own judgment rather than allowing AI to dictate the care process.

### Healthcare provider well-being

How the Japanese Association of Rehabilitation Nutrition supports the accumulation of capital, happiness, and well-being of healthcare providers practicing rehabilitation nutrition could determine the future development of both the association and the research field. While self-care encompassing physical, psychological, social, spiritual, and technological aspects forms the foundation of healthcare provider well-being, training sessions on self-care methods are needed. For example, online training sessions can serve as a vital platform for individuals to evaluate and reflect on their exercise and dietary habits, ideal weight, social participation, mental health, sense of purpose, and AI use.

### Global dissemination

Based on evidence directly generated from clinical implementation in Japan, the active dissemination of Japan’s established knowledge and clinical practices in rehabilitation nutrition provides a unique opportunity to effectively address global health challenges ^(14)^. The development of the International Association of Rehabilitation Nutrition and the launch of an English-language journal dedicated to rehabilitation nutrition would further support global dissemination of rehabilitation nutrition.

## Conclusion

Rehabilitation nutrition 3.0 is a comprehensive and individualized approach that maximizes the functioning and well-being of both individuals and healthcare providers by leveraging physical, psychological, social, spiritual, and technological aspects. Building upon the foundations of rehabilitation nutrition 2.0, rehabilitation nutrition 3.0 is expected to evolve toward greater comprehensiveness and personalization. By integrating insights from the humanities, social sciences, and information science alongside medicine and nutrition, this approach transcends a purely physical focus. This position paper serves as both a road map and a compass for guiding the field until the emergence of rehabilitation nutrition 4.0 in the 2030s.

## Article Information

### Acknowledgments

We solicited public comments from members of the Japanese Association of Rehabilitation Nutrition from September 4 to 18, 2025. One respondent provided valuable comments that we incorporated into the manuscript. We would like to express our deepest gratitude to all those who provided public comments. We confirm that the generative artificial intelligence tools Gemini and DeepL were used solely for editing and refining the English language and clarity of the final manuscript.

### Author Contributions

Hidetaka Wakabayashi: conceptualization, data curation, formal analysis, funding acquisition, investigation, methodology, project administration, resources, software, and writing – original draft. Keisuke Maeda: data curation, formal analysis, methodology, and writing – review and editing. Shinjiro Miyazaki: methodology and writing – review and editing. Mariko Nakamichi: methodology and writing – review and editing. Dai Fujiwara: project administration, supervision, and writing – review and editing. All authors approved the final manuscript and agreed to be accountable for all aspects of the work and to ensure that questions related to the accuracy or integrity of any part of the work are appropriately investigated and resolved.

### Conflicts of Interest

Hidetaka Wakabayashi received a speaking fee from Otsuka Pharmaceutical Factory. Keisuke Maeda received a speaking fee from The Nisshin OilliO Group, Ltd. The other authors have no conflicts of interest to declare.

### Footnote

This is the secondary English version of the original Japanese manuscript for “Rehabilitation nutrition 3.0—The dissemination and evolution of comprehensive and individualized rehabilitation nutrition: A position paper by the Japanese Association of Rehabilitation Nutrition” published in the Journal of the Japanese Association of Rehabilitation Nutrition in 2026;10(1):89-96. The authors have obtained permission for secondary publication from the Editor of the Journal of the Japanese Association of Rehabilitation Nutrition.

## References

[1] Wakabayashi H. Rehabilitation nutrition for PTs, OTs, and STs: how nutrition care transforms rehabilitation. Tokyo (Japan): Ishiyaku Publishers, Inc; 2010. 120 p. Japanese.

[2] Wakabayashi H. Handbook of rehabilitation nutrition. Tokyo (Japan): Ishiyaku Publishers, Inc; 2010. 292 p. Japanese.

[3] Wakabayashi H. Rehabilitation nutrition in general and family medicine. J Gen Fam Med. 2017;18(4):153-4. 10.1002/jgf2.116 PMC5675160 29264014

[4] Nagano A, Nishioka S, Wakabayashi H. Rehabilitation nutrition for iatrogenic sarcopenia and sarcopenic dysphagia. J Nutr Health Aging. 2019;23(3):256-65. 10.1007/s12603-018-1150-1 PMC12280483 30820514

[5] Nishioka S, Aragane H, Suzuki N, et al. Clinical practice guidelines for rehabilitation nutrition in cerebrovascular disease, hip fracture, cancer, and acute illness: 2020 update. Clin Nutr ESPEN. 2021;43:90-103. 10.1016/j.clnesp.2021.02.018 34024570

[6] Fujishima I, Fujiu-Kurachi M, Arai H, et al. Sarcopenia and dysphagia: position paper by four professional organizations. Geriatr Gerontol Int. 2019;19(2):91-7. 10.1111/ggi.13591 30628181

[7] Sato S, Miyazaki S, Tamaki A, et al. Respiratory sarcopenia: a position paper by four professional organizations. Geriatr Gerontol Int. 2023;23(1):5-15. 10.1111/ggi.14519 PMC11503586 36479799

[8] Inoue T, Iida Y, Takahashi K, et al. Nutrition and Physical therapy: a position paper by the physical therapist section of the Japanese Association of Rehabilitation Nutrition (Secondary Publication). JMA J. 2022;5(2):243-51. 10.31662/jmaj.2021-0201 PMC9090552 35611222

[9] Inoue T, Takeuchi I, Iida Y, et al. Disease-specific nutritional physical therapy: a position paper by the Japanese Association of Rehabilitation Nutrition (Secondary Publication). JMA J 2022;5(2):252-62. 10.31662/jmaj.2021-0202 PMC9090541 35611233

[10] Wakabayashi H. Rehabilitation pharmacotherapy: a combination of rehabilitation and pharmacotherapy. J Gen Fam Med. 2018;19(2):43-4. 10.1002/jgf2.163 PMC5867163 29600126

[11] Kose E, Wakabayashi H. Rehabilitation pharmacotherapy: a scoping review. Geriatr Gerontol Int. 2020;20(7):655-63. 10.1111/ggi.13975 32691925

[12] Wakabayashi H. Triad of rehabilitation, nutrition, and oral management for sarcopenic dysphagia in older people. Geriatr Gerontol Int. 2024;24 Suppl 1:397-9. 10.1111/ggi.14651 37577770

[13] Wakabayashi H. The rehabilitation nutrition oral care process: implementing the triad of rehabilitation, nutrition, and oral management. J Gen Fam Med. 2025;26(2):114-5. 10.1002/jgf2.763 PMC11890041 40061400

[14] Yoshimura Y, Wakabayashi H, Matsumoto A, et al. A triad strategy of rehabilitation, nutrition, and oral management for malnutrition, sarcopenia, and frailty in super-aged societies. Nutrition. 2026;142:112959. 10.1016/j.nut.2025.112959 41092813

[15] Wakabayashi H, Mori T, Nishioka S, et al. Psychological aspects of rehabilitation nutrition: a position paper by the Japanese Association of Rehabilitation Nutrition (secondary publication). J Gen Fam Med. 2024;25(1):1-9. 10.1002/jgf2.668 PMC10792333 38240004

[16] Wakabayashi H. Positive psychology and rehabilitation nutrition. J Gen Fam Med. 2022;23(5):293-4. 10.1002/jgf2.567 PMC9444013 36093225

[17] Fujiwara D, Wakabayashi H, Maeda K. Rehabilitation nutrition in the social context: perspectives and practices linking sarcopenia, frailty, nutritional disorders, and the social determinants of health—a position paper by the Japanese Association of Rehabilitation Nutrition. J Jpn Assoc Rehabil Nutr. 2026;10(1):80-88. Japanese.

[18] Wakabayashi H, Mori T, Nishioka S, et al. Consideration of spiritual aspects in rehabilitation nutrition: position paper by the Japanese Society for Rehabilitation Nutrition. J Jpn Assoc Rehabil Nutr. 2025;9(2):235-42. Japanese.

[19] Wakabayashi H, Yoshimura Y, Maeda K, et al. Goal setting for nutrition and body weight in rehabilitation nutrition: position paper by the Japanese Association of Rehabilitation Nutrition (secondary publication). J Gen Fam Med. 2022;23(2):77-86. 10.1002/jgf2.509 PMC8888801 35261854

[20] Nakahara S, Takasaki M, Abe S, et al. Aggressive nutrition therapy in malnutrition and sarcopenia. Nutrition. 2021;84:111109. 10.1016/j.nut.2020.111109 33453622

[21] Nishioka S, Nakahara S, Takasaki M, et al. The concept of aggressive nutrition therapy and clinical indication: a position paper. Clin Nutr ESPEN. 2022;52:322-30. 10.1016/j.clnesp.2022.09.013 36513472

[22] Wakabayashi H, Maeda K, Momosaki R, et al. Diagnostic reasoning in rehabilitation nutrition: position paper by the Japanese Association of Rehabilitation Nutrition (secondary publication). J Gen Fam Med. 2022;23(4):205-16. 10.1002/jgf2.549 PMC9249927 35800646

[23] Noetel M, Sanders T, Gallardo-Gómez D, et al. Effect of exercise for depression: systematic review and network meta-analysis of randomised controlled trials. BMJ. 2024;384:e075847. 10.1136/bmj-2023-075847 PMC10870815 38355154

[24] Solmi M, Basadonne I, Bodini L, et al. Exercise as a transdiagnostic intervention for improving mental health: an umbrella review. J Psychiatr Res. 2025;184:91-101. 10.1016/j.jpsychires.2025.02.024 40043589

[25] Hu W, Mao H, Guan S, et al. Systematic review and meta-analysis of the association between malnutrition and risk of depression in the elderly. Alpha Psychiatry. 2024;25(2):183-9. 10.5152/alphapsychiatry.2024.231336 PMC11117414 38798803

[26] Jokela M, Laakasuo M. Obesity as a causal risk factor for depression: systematic review and meta-analysis of Mendelian Randomization studies and implications for population mental health. J Psychiatr Res. 2023;163:86-92. 10.1016/j.jpsychires.2023.05.034 37207436

[27] Fabricatore AN, Wadden TA, Higginbotham AJ, et al. Intentional weight loss and changes in symptoms of depression: a systematic review and meta-analysis. Int J Obes. 2011;35(11):1363-76. 10.1038/ijo.2011.2 PMC3139753 21343903

[28] Ghaemi S, Zeraattalab-Motlagh S, Jayedi A, et al. The effect of vitamin D supplementation on depression: a systematic review and dose-response meta-analysis of randomized controlled trials. Psychol Med. 2024;54(15):3999-4008. 10.1017/S0033291724001697 PMC11650176 39552387

[29] da Silva LEM, de Santana MLP, Costa PRF, et al. Zinc supplementation combined with antidepressant drugs for treatment of patients with depression: a systematic review and meta-analysis. Nutr Rev. 2021;79(1):1-12. 10.1093/nutrit/nuaa039 32885249

[30] Saghafian F, Hajishafiee M, Rouhani P, et al. Dietary fiber intake, depression, and anxiety: a systematic review and meta-analysis of epidemiologic studies. Nutr Neurosci. 2023;26(2):108-26. 10.1080/1028415X.2021.2020403 36692989

[31] Asad A, Kirk M, Zhu S, et al. Effects of prebiotics and probiotics on symptoms of depression and anxiety in clinically diagnosed samples: systematic review and meta-analysis of randomized controlled trials. Nutr Rev. 2025;83(7):e1504-20. 10.1093/nutrit/nuae177 PMC12166186 39731509

[32] Sulaiman NNY, Mohamad Nizam NB, Mohd Noor NA, et al. An updated systematic review and appraisal of the pathophysiologic mechanisms of probiotics in alleviating depression. Nutr Neurosci. 2025;28(12):1564-84. 10.1080/1028415X.2025.2531357 40669008

[33] Li J, Wang H, Li M, et al. Effect of alcohol use disorders and alcohol intake on the risk of subsequent depressive symptoms: a systematic review and meta-analysis of cohort studies. Addiction. 2020;115(7):1224-43. 10.1111/add.14935 31837230

[34] Dai S, Wellens J, Yang N, et al. Ultra-processed foods and human health: an umbrella review and updated meta-analyses of observational evidence. Clin Nutr. 2024;43(6):1386-94. 10.1016/j.clnu.2024.04.016 38688162

[35] Lane MM, Gamage E, Du S, et al. Ultra-processed food exposure and adverse health outcomes: umbrella review of epidemiological meta-analyses. BMJ. 2024;384:e077310. 10.1136/bmj-2023-077310 PMC10899807 38418082

[36] Becker JC, Hartwich L, Haslam SA. Neoliberalism can reduce well-being by promoting a sense of social disconnection, competition, and loneliness. Br J Soc Psychol. 2021;60(3):947-65. 10.1111/bjso.12438 33416201

[37] Funao H, Momosaki R, Tsujikawa M, et al. Application of virtual reality to home-visit rehabilitation for patients with chronic musculoskeletal pain: a single-group pre-post comparison study. Cureus. 2025;17(3):e80386. 10.7759/cureus.80386 PMC11984008 40213767

[38] Deng W, Zeng XF, Zhang LY, et al. The management of sarcopenic dysphagia: a multidisciplinary approach leveraging emerging technologies. Aging Dis. 2024;16(5):2752-69. 10.14336/AD.2024.0741 PMC12339130 39500356

[39] Brinsley J, O’Connor EJ, Singh B, et al. Effectiveness of digital lifestyle interventions on depression, anxiety, stress, and well-being: systematic review and meta-analysis. J Med Internet Res. 2025;27:e56975. 10.2196/56975 PMC11969127 40112295

[40] Getie A, Ayenew T, Amlak BT, et al. Global prevalence and contributing factors of nurse burnout: an umbrella review of systematic review and meta-analysis. BMC Nurs. 2025;24(1):596. 10.1186/s12912-025-03266-8 PMC12108038 40420259

[41] Hodkinson A, Zhou A, Johnson J, et al. Associations of physician burnout with career engagement and quality of patient care: systematic review and meta-analysis. BMJ. 2022;378:e070442. 10.1136/bmj-2022-070442 PMC9472104 36104064

[42] Li LZ, Yang P, Singer SJ, et al. Nurse burnout and patient safety, satisfaction, and quality of care: a systematic review and meta-analysis. JAMA Netw Open. 2024;7(11):e2443059. 10.1001/jamanetworkopen.2024.43059 PMC11539016 39499515

[43] Badriyah FL, Suarilah I, Tarihoran DETAU, et al. Effectiveness of cognitive behavioral therapy in reducing psychological distress in nurses: a systematic review and meta-analysis of experimental studies. Nurs Health Sci. 2025;27(1):e70036. 10.1111/nhs.70036 39821678

[44] Dou J, Lian Y, Lin L, et al. Effectiveness of mindfulness-based interventions on burnout, resilience and sleep quality among nurses: a systematic review and meta-analysis of randomized controlled trials. BMC Nurs. 2025;24(1):739. 10.1186/s12912-025-03101-0 PMC12210539 40598019

[45] Jones KF, Pryor J, Care-Unger C, et al. “Spirituality is everybody’s business”: an exploration of the impact of spiritual care training upon the perceptions and practice of rehabilitation professionals. Disabil Rehabil. 2022;44(8):1409-18. 10.1080/09638288.2020.1820586 32976734

[46] Wang X, Zhou Y, Zhou G. The application and ethical implication of generative AI in mental health: systematic review. JMIR Ment Health. 2025;12:e70610. 10.2196/70610 PMC12254713 40577783

[47] Ratti E, Morrison M, Jakab I. Ethical and social considerations of applying artificial intelligence in healthcare-a two-pronged scoping review. BMC Med Ethics. 2025;26(1):68. 10.1186/s12910-025-01198-1 PMC12107984 40420080

